# Comparison of 3- and 20-Gradient Direction Diffusion-Weighted Imaging in a Clinical Subacute Cohort of Patients with Transient Ischemic Attack: Application of Standard Vendor Protocols for Lesion Detection and Final Infarct Size Projection

**DOI:** 10.3389/fneur.2017.00691

**Published:** 2017-12-18

**Authors:** Inger Havsteen, Christian Ovesen, Lasse Willer, Janus Damm Nybing, Karen Ægidius, Jacob Marstrand, Per Meden, Sverre Rosenbaum, Marie Norsker Folke, Hanne Christensen, Anders Christensen

**Affiliations:** ^1^Department of Radiology, Bispebjerg Hospital, Copenhagen University Hospital, Copenhagen, Denmark; ^2^Department of Neurology, Bispebjerg Hospital, Copenhagen University Hospital, Copenhagen, Denmark

**Keywords:** transient ischemic attack, diffusion-weighted imaging, diffusion tensor imaging, infarction, magnetic resonance imaging

## Abstract

**Objective:**

Diffusion tensor imaging may aid brain ischemia assessment but is more time consuming than conventional diffusion-weighted imaging (DWI). We compared 3-gradient direction DWI (3DWI) and 20-gradient direction DWI (20DWI) standard vendor protocols in a hospital-based prospective cohort of patients with transient ischemic attack (TIA) for lesion detection, lesion brightness, predictability of persisting infarction, and final infarct size.

**Methods:**

We performed 3T-magnetic resonance imaging including diffusion and T2-fluid attenuated inversion recovery (FLAIR) within 72 h and 8 weeks after ictus. Qualitative lesion brightness was assessed by visual inspection. We measured lesion area and brightness with manual regions of interest and compared with homologous normal tissue.

**Results:**

117 patients with clinical TIA showed 78 DWI lesions. 2 lesions showed only on 3DWI. No lesions were uniquely 20DWI positive. 3DWI was visually brightest for 34 lesions. 12 lesions were brightest on 20DWI. The median 3DWI lesion area was larger for lesions equally bright, or brightest on 20DWI [median (IQR) 39 (18–95) versus 18 (10–34) mm^2^, *P* = 0.007]. 3DWI showed highest measured relative lesion signal intensity [median (IQR) 0.77 (0.48–1.17) versus 0.58 (0.34–0.81), *P* = 0.0006]. 3DWI relative lesion signal intensity was not correlated to absolute signal intensity, but 20DWI performed less well for low-contrast lesions. 3DWI lesion size was an independent predictor of persistent infarction. 3-gradient direction apparent diffusion coefficient areas were closest to 8-week FLAIR infarct size.

**Conclusion:**

3DWI detected more lesions and had higher relative lesion SI than 20DWI. 20DWI appeared blurred and did not add information.

**Clinical Trial Registration:**

http://www.clinicaltrials.gov. Unique Identifier NCT01531946.

## Introduction

The risk of recurrent stroke after transient ischemic attack (TIA) is considerable (1), and clinical scores, as the ABCD2, have been validated and implemented in the guidelines (2). Recently, scores combining the clinical findings with diffusion-weighted imaging (DWI) lesion presence on magnetic resonance imaging (MRI), as, e.g., the ABCD3-I score, have been shown to improve subsequent stroke risk estimates in TIA patients (3–5).

Among patients with a clinical TIA diagnosis, DWI lesions are reported in 25–50% (4, 6–11), but an unexplained sevenfold variation in DWI positivity exists in literature (12). Rates are halved in populations with high stroke awareness, and easy-access high-volume TIA clinics (13). Large studies report lower rates compared with small studies, and time from ictus may influence the numbers underlining the instability of DWI positivity (12, 14).

Fast 3-gradient direction DWI (3DWI) shows acute ischemic lesions few minutes after ictus (15), and final lesion size is considered stationary after 7–30 days (16, 17). DWI lesions are heterogeneous, possibly as sign of varying degree of tissue damage, location, and recovery after reperfusion (18–20). This influences the subsequent extent of infarction on follow-up examinations (21). Diffusion tensor imaging (DTI) uses an increased number of diffusion encoding gradient directions and may depict brain microarchitecture including complex white matter tracts crossings even on subvoxel level (22). Despite postprocessing challenges, the technique is robust (23) and is widely used clinically (24, 25). The more time-consuming DTI has been reported to be of value in assessing the presence and degree of brain ischemia in acute stroke and TIA (26–29) and aids the identification of small and low-contrast lesions, even beyond the capability of standard 3DWI (30). DTI may be able to provide more homogeneous ischemia detection in the subacute phase, possibly with better correlation to the extent of the final lesion, and may thus help to better define patients with transitory symptoms with infarction (TSI) (31). We hypothesized that higher numbers of diffusion encoding gradients could add to qualitative lesion identification and delineation in a clinical setting.

However, any MRI protocol investigating acute or subacute brain ischemia must balance the diagnostic gain from longer sequences with scan time limits dictated primarily by the presence of critical motion artifacts (32, 33). Motion artifact rates rise with pathology presence (34), and in inpatient and emergency department settings (35). Hence, the best trade-off remains to be investigated in a clinical setting. We hypothesized that 20DWI would show equal or better ischemia detection and characterization for small lesions, than standard 3DWI. We aimed to compare fast standard vendor protocols for conventional 3DWI with 20-gradient direction DWI (20DWI), concerning lesion detection and characterization in subacute patients with TIA in a clinical setting. Specifically we compared 3- and 20DWI sequences for initial DWI lesion detection, measured and qualitative lesion brightness, and their ability to predict lasting infarction 8 weeks after ictus.

## Materials and Methods

We investigated a prospective patient cohort with clinical TIA included February 2012–December 2014. The study was approved by the National Committee of Biomedical Research Ethics (H-1-2011-75, ClinicalTrials.gov Identifier NCT01531946). Patients were included after informed consent, in accordance with the Declaration of Helsinki.

### Population and Procedures

On admission, a senior consultant stroke neurologist clinically evaluated the patients. We defined TIA as focal neurological deficit with resolution (National Institutes of Health Stroke Scale 0) within 24 h, attributed to a vascular ischemic incidence.

Exclusion criteria were contraindications to MRI, non-TIA diagnosis, herein exclusion of patients treated with thrombolytics, and severe comorbidity likely to precluding follow-up. Included patients received MRI at two time points: routine brain ischemia imaging supplemented with 20DWI within 72 h, and at 8-week follow-up standard 3DWI and T2-fluid attenuated inversion recovery (FLAIR) to assess for new lesions.

We recorded symptoms, symptom duration, vascular risk factors, and ABCD2 and anamnestic factors including prior stroke, TIA or myocardial infarction (MI), angina pectoris, peripheral arterial disease, diabetes, or depression. We defined hypertension as preadmission use of antihypertensive medication, or hypertension diagnosis in the outpatient clinic. Atrial fibrillation was identified through medical history, admission 12-lead ECG, in-hospital telemetry (24–48 h) or subsequent outpatient cardiac follow-up. Hypercholestrolemia was total plasma cholesterol over 5.0 mmol/L or statin treatment. Medical history or HbA1c > 6.5% defined diabetes. Smoking was present or prior smoking, and alcohol overuse was weekly alcohol intake over 252 g for males and 168 g for females. First degree relative with stroke or MI were recorded as hereditary risk factors.

Upon arrival, patients underwent non-contrast head computed tomography and standard medical treatment, that is, admission to a specialized stroke ward, acute administration of two platelet-inhibiting agents, and 24 h of electrocardiography observation. After the acute treatment, standard post-TIA workup included subacute MRI, risk-factor modification, and carotid ultrasound. CT-angiography or transcranial Doppler was not part of the protocol.

### Image Acquisition

We performed 3T-MRI (Siemens Magnetom Verio, Siemens, Erlangen, Germany) with a 32-channel head coil (Siemens, Erlangen, Germany).

3-Gradient direction DWI was Siemens’ single-shot spin-echo diffusion echo-planar imaging protocol with 220-mm field of view (FOV), 25 4-mm axial 0-mm gap slices, *b*-values 0 and 1,000 s/mm^2^ along 3 orthogonal directions; repetition time (TR)/echo time (TE) 6,600/100 ms, acceleration factor *R* 2, in plane resolution for 3DWI and 3-gradient direction apparent diffusion coefficient (ADC) was 1.1 mm × 1.1 mm, 4 averages, bandwidth 1,002 Hz/Px, and scan time 2:07 min:s.

20DWI was Siemens’ single-shot echo-planar imaging protocol with 230 mm FOV, 25 4-mm axial 0-mm gap slices, TR/TE 3,600/95 ms, acceleration factor *R* 2, in plane resolution for 20DWI and 20ADC was 1.8 mm × 1.8 mm, *b*-values 0, and 1,000 s/mm^2^, 3 averages, bandwidth 1,502 Hz/Px, and scan time 4:39 min:s.

The T2-FLAIR protocol was 240-mm FOV, 27 4-mm axial 0-mm gap slices, TR/TE 6,500/133 ms, TI 2,134 ms, acceleration factor *R* 2, in plane resolution 0.9 mm × 0.9 mm, and scan time 3:32 min:s.

The 20DWI protocol’s scan parameters used, including the number of directions, the lower matrix size, and averages were vendor’s best compromise for acceptable image quality and scan time. Vendor’s noise reduction postprocessing algorithm in 3ADC and 20DWI and 20ADC nulls signal from air. Further details on postprocessing and their impact on signal intensity (SI) were not available. The baseline protocol also included perfusion sequences.

### Image Interpretation

We performed the study in a clinical setting using the PACS only, without external software. One neuroradiologist (IH) systematically evaluated all scans according to a predefined chart. The radiologist was informed of side and nature of neurological symptoms through the PACS referral.

**Figure 1:**
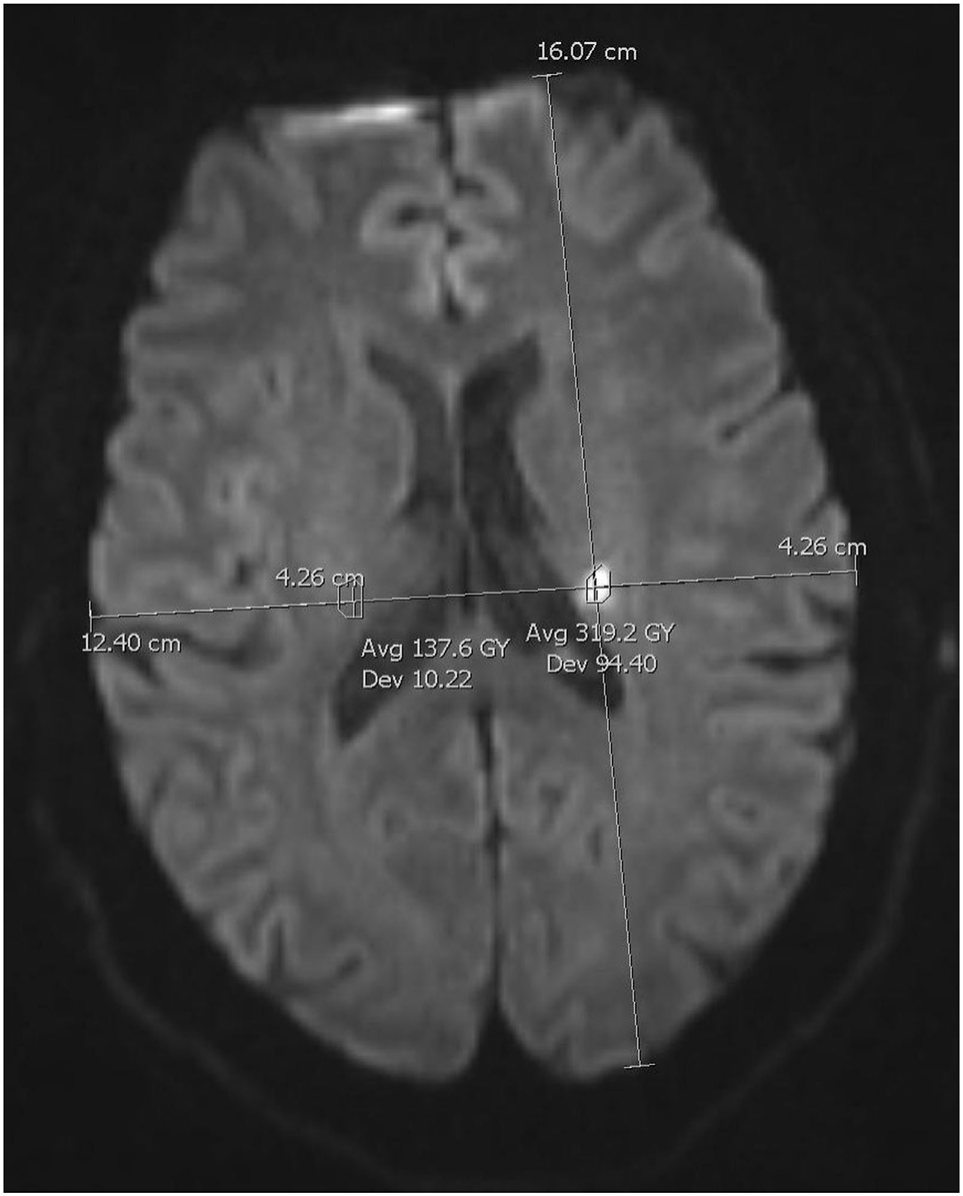
Regions of interest measurements. Co-registration method for lesion size and signal intensity on 3-gradient direction DWI.

We co-registered lesions describing their positions with two perpendicular intrathecal diameters to ascertain lesion localization between sequences and baseline, and 8-week MRI (Figure 1). DWI hyperintensities were compared with ADC, and initial T2-FLAIR images for ADC confirmation, and to avoid T2 shine through from chronic infarctions or leukoaraiosis. Two separate lesions should not have confluent gliosis. We defined 8-week infarction as presence of T2-hyperintensity, or atrophy in the area of initial DWI lesion at 8-week follow-up. After all radiological and clinical data were collected, we investigated if symptoms matched lesion site, under supervision of a senior stroke neurologist consultant (HC).

### Lesion Size Estimate

We defined lesion size as the largest lesion area measured by manual regions of interest (ROIs), delineating lesion periphery on a single slice in 3DWI, 3ADC, 20DWI, 20ADC, initial T2-FLAIR, and 8-week T2-FLAIR (30).

### Qualitative Lesion Brightness and Measured SI Differences

**Figure 2:**
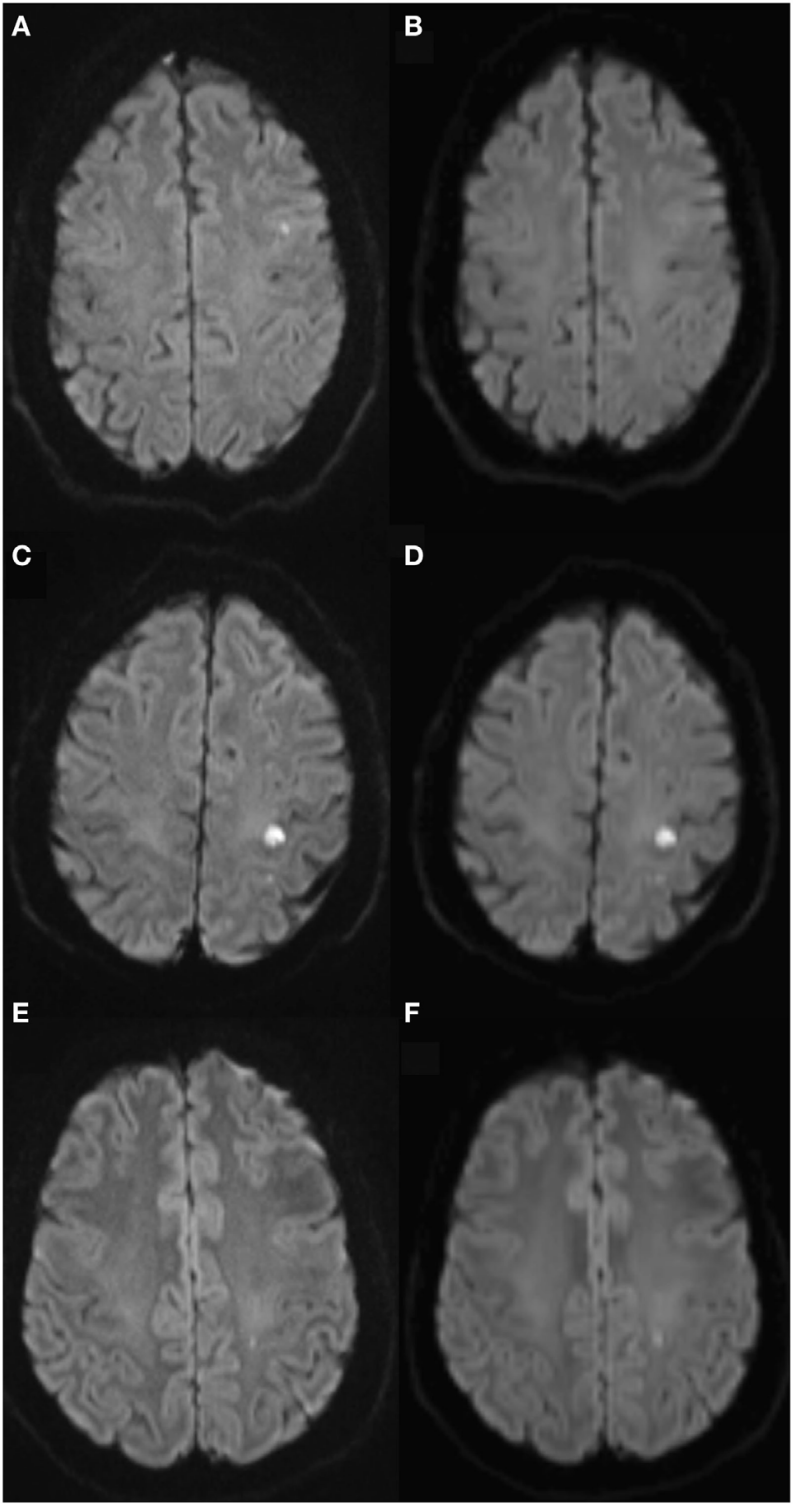
Qualitative lesion brightness on 3- and 20-gradient diffusion imaging. Diffusion-weighted imaging (DWI) lesion shows on 3-gradient DWI (3DWI) **(A)**, 20-gradient DWI **(B)** shows no certain lesion, but blur. Panels **(C,D)** equal lesion brightness on 3DWI **(C)** and 20DWI **(D)**. Panels **(E,F)** lesion brightest on 20DWI (**F)**.

We scored lesion brightness on 3- and 20DWI and assessed if a lesion was more distinct on either or equal, blinded to other findings (Figure 2).

We assessed lesions according to the “hot spot” method (36, 37). For lesions visible on both 3- and 20DWI, we measured lesion size and SI. We drew an ROI around the lesion’s area, and a second ROI covering a homologous area in the contralateral hemisphere, avoiding areas of chronic infarction, leukoaraiosis or cerebrospinal fluid. We used manual ROIs (Figure 1).

For 3DWI and 20DWI sequences, we calculated a relative signal intensity (rSI) difference between ipsilateral (i) infarcted and contralateral (c) normal tissue: rSI = (SI_i_ − SI_c_)/SI_c_. We compared the sequences’ relative signal variation using medians and interquartile ranges (IQRs).

To compare sequences we calculated the sequences’ difference in relative signal intensity compared with the standard 3DWI’s relative signal intensity: %SI = 100*(rSI_20_ − rSI_3_)/rSI_3_ (30).

### Statistics

Data are shown as frequencies, medians with IQR, or odds ratio (OR) with 95% confidence intervals (95% CI) as appropriate. We compared categorical data using the Fisher’s exact test, and continuous and ordinal scale data using the Mann–Whitney *U* test. Proportions of repeated measurements were analyzed using McNemar’s test. Lesion area measurement on the different sequences was compared using the Bland–Altman technique and calculated the median deviation and the mean bias. We performed logistic regression analysis and used Wald test for *P*-values. We tested for correlations using Spearman’s rho. We considered *P*-values less than 0.05 significant. For statistical computation we used R (version 3.2.0), 2015 The R Foundation for Statistical Computing, Vienna, Austria—http://www.R-project.org/ and SPSS (version 22.0) statistical software, IBM Corporation, Armonk, NY, USA. In a 10% sample (patients born 4th, 14th, and 24th day in any month), we calculated Cohen’s kappa for intraobserver variation of qualitative data using two readings with 3 months’ interval, and found substantial intraobserver agreement (kappa 0.80).

## Results

**Figure 3:**
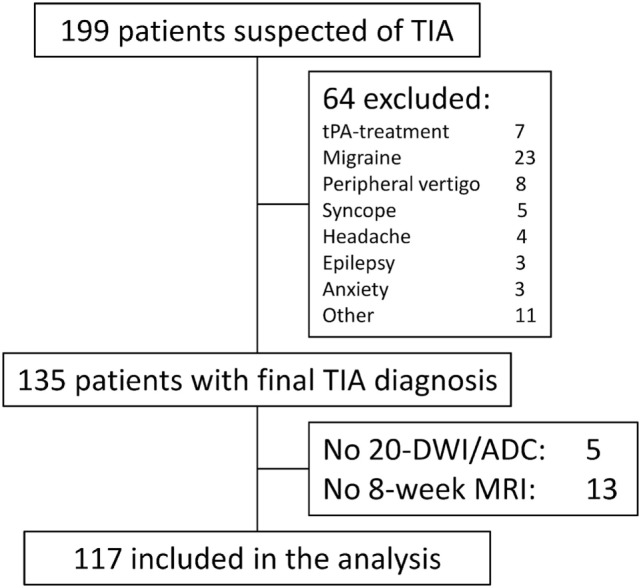
STROBE diagram of patient in- and exclusion. Other contained patients with peripheral nerve compression (2), ophthalmological symptoms (2), trigeminal neuralgy (1), normal pressure hydrocephalus (1), hyperventilation (1), paresthesia secondary to anemia (1), peripheral extremity embolus (1), food poisoning (1), and secondary refusal (1). Reasons for lack of 20DWI/apparent diffusion coefficient (ADC) were technician error (3), incomplete examination due to restless patient (1), and technical error (1).

Among 199 consenting and included patients, 64 patients were later excluded, due to final non-TIA diagnosis, according to treating stroke neurologist. Further 18 patients were excluded, due to unavailable 20DWI sequence, or as they declined the 8-week MRI (Figure 3). The final cohort contained 117 patients with TIA with 149 clinical or radiological events. 46 patients showed 78 diffusion lesions. Patient characteristics are presented in Table 1. The median (IQR) time from ictus to initial MRI was 32 (24–57) h.

**Table 1:** Patient characteristics.

All patients	117
Female sex, *n*	51 (44%)
Age, median (IQR)	65 (55–71)
**Medical history**
Prior stroke	21 (18%)
Prior TIA	12 (10%)
Prior MI	8 (7%)
Atrial fibrillation	12 (10%)
Hypertension	56 (48%)
Diabetes	16 (14%)
Depression	12 (10%)
Current smoking	39 (34%)
Alcohol overuse	11 (10%)
Antiplatelet use	40 (34%)
Warfarin use	1 (1%)
**Index stroke**
ABCD2, median (IQR)	4 (3–5)
Duration of symptoms (min)
<60	57 (49%)
>60	60 (51%)
TOAST etiology
Small vessels	47 (40%)
Large vessels	25 (21%)
Cardiogenic	18 (15%)
Multiple possible etiologies	27 (23%)
TTS (h)	32 (24–57)
TTF (days)	56 (55–60)

*Numbers are frequency (%) unless otherwise indicated*.

*TIA, transient ischemic attack; MI, myocardial infarction; IQR, interquartile range; TTS, time to scan; TTF, time to follow-up*.

We lost 18 patients for analysis (13%, 18/135). The five patients with no 20DWI/ADC were diffusion positive. Among the 13 patients with no 8-week MRI, 5 patients were diffusion positive. Thus, among the 135 patients with clinical TIA, 56 patients (41%) were diffusion positive. This proportion is not significantly different from the group included for analysis (*P* = 0.71).

### Lesion Detection

On initial scan 78 (52%) lesions were 3- and 76 (51%) 20DWI positive (*P* = 0.500, Table 2). 51 (34%) lesions showed hyperintensity or atrophy on 8-week FLAIR. Hereof 50 lesions were initially DWI positive. One initially DWI-negative 8-week hyperintense FLAIR lesion was found in a patient with small vessel disease pattern, which may have been invisible early after reperfusion (14), or a silent intercurrent infarction.

**Table 2:** Baseline lesion areas (cm^2^) for diffusion-weighted imaging (DWI)-positive lesions.

Lesions	3-Gradient direction DWI	20DWI*	3ADC	20ADC*	T2-initial fluid attenuated inversion recovery (inFLAIR)
*N*, all	78	76	71	70	63
Range	0.03–5.88	0.05–5.38	0.03–5.07	0.03–5.31	0.05–6.03
Median [interquartile range (IQR)]	0.26 (0.11–0.59)	0.34 (0.19–0.79)	0.23 (0.14–0.60)	0.24 (0.12–0.63)	0.31 (0.15–0.80)
*N*, scar	50 (64%)	50 (66%)	49 (69%)	48 (69%)	50 (79%)
Range, scar	0.05–5.88	0.06–5.38	0.03–5.07	0.05–5.31	0.05–6.03
Range, no scar	0.03–1.10	0.05–1.05	0.03–2.17	0.03–1.55	0.04–1.56
Median (IQR), scar	0.41 (0.13–0.86)	0.53 (0.26–1.07)	0.34 (0.16–1.00)	0.38 (0.18–0.86)	0.37 (0.18–0.89)
Median (IQR), no scar	0.17 (0.08–0.22)	0.21 (0.10–0.27)	0.17 (0.07–0.23)	0.12 (0.08–0.18)	0.20 (0.08–0.89)
*P*^a^	*P* < 0.0001	*P* < 0.0001	*P* = 0.001	*P* < 0.0001	*P* < 0.0001

*117 patients with 149 events completed 3- and 20DWI sequences and 8-week (8w) magnetic resonance imaging*.

*^a^Mann–Whitney U test for 8-week scar versus no scar*.

*^3^1 lesion appeared only on 8w-FLAIR (DWI negative), it is in these data but is probably an intercurrent lesion*.

*20DWI/apparent diffusion coefficient (ADC) data were unavailable for 5 patients with 6 lesions.

*In, initial*.

Two lesions were only visible on 3DWI, they were small, measuring 0.03 and 0.11 cm^2^. Both lesions showed no ADC confirmation and no infarction on 8-week FLAIR. No lesions were uniquely 20DWI or -ADC positive.

### Lesion Size

Lesions were slightly, non-significantly, smaller on 3DWI than 20DWI [median (IQR) 0.29 (0.11–0.72) cm^2^ versus 0.35 (0.20–0.80) cm^2^, *P* = 0.26].

### Qualitative Lesion Brightness

Among events 3DWI was qualitatively most distinct in 34 lesions (22%), 12 lesions (8%) were most conspicuous on 20DWI (Table 3). 32 lesions (22%) were equally conspicuous on 3- and 20DWI. The median (IQR) 3DWI lesion area was larger for lesions brightest on 20DWI, or equally bright compared with 3DWI-brightest lesions [39 (18–95) versus 18 (10–34) mm^2^, *P* = 0.007]. Among patients with diffusion lesions, the probability of persistent infarction was significantly higher for lesions equally positive or brightest on 20DWI, compared with lesions brightest on 3DWI (crude OR 3.83, 95% CI: 1.44–10.14). In general, the predictive value toward persistent infarction was the same for patients with lesions on 3DWI or 20DWI (Table 4). The negative predictive value (both 0.99) was high. The positive predictive values (0.64 and 0.66) were fair for both. The strongest positive predictive value for infarction was, if either the 20DWI lesion was most prominent, or if the lesion was equally bright on 3DWI and 20DWI (PPV 0.77). To investigate, if the association between lesions most bright on 20DWI (or equal to 3DWI) and persistent infarction signs was confounded by the size of the lesion, we adjusted for lesion areas measured on 3DWI. Lesion area size emerged as an independent predictor of lasting infarction, independent of perceived brightness on any sequence, and perceived brightness lost its predictive capability. 20DWI lesions often appeared blurred (Figure 2), probably due to lower resolution.

**Table 3:** Qualitative brightness and probability of 8-week infarction among 149 clinical or radiological transient ischemic attack events.

Most distinct sequence	*N* (%)	*P* of infarction (%)	Crude OR	Adjusted OR
Brightest on 3DWI	34 (23)	47	1.00	1.00
Brightest on 20DWI	12 (8)	75	3. 38 (0.78–14.68)	3.55 (0.74–7.10)
Equally positive	32 (22)	78	4.02 (1.37–11.77)	2.38 (0.72–17.10)
Negative on both	71 (48)	1	–	–

*P of infarction is the probability of persistent infarction within each brightness category. Crude OR is the crude odds ratio for persistent infarction within diffusion-weighted imaging-positive lesions, reference category is lesions brightest on 3-gradient direction DWI (3DWI). Adjusted OR is the crude OR adjusted for 3DWI lesion size*.

**Table 4:** Binary classification measures for lasting infarction for qualitatively most distinct sequence.

Most distinct sequence	Sensitivity	Specificity	PPV	NPV	Accuracy
3DWI lesion	0.98	0.71	0.64	0.99	0.52
20DWI lesion	0.98	0.73	0.66	0.99	0.70
3DWI brightest + equal	0.80	0.74	0.62	0.88	0.77
20DWI brightest + equal	0.67	0.90	0.77	0.84	0.82

*Lesions appearing subjectively most distinct on either 3- direction DWI (3DWI) or 20-gradient DWI (20DWI) sequence: sensitivity, specificity, positive (PPV) and negative predictive value (NPV), and accuracy for 8-week scarring in 149 lesions. 51 lesions show 8-week scar. Equal describes DWI-positive lesions that appear equally bright on 3- and 20DWI*.

### Lesion Contrast as Measured SI

**Figure 4:**
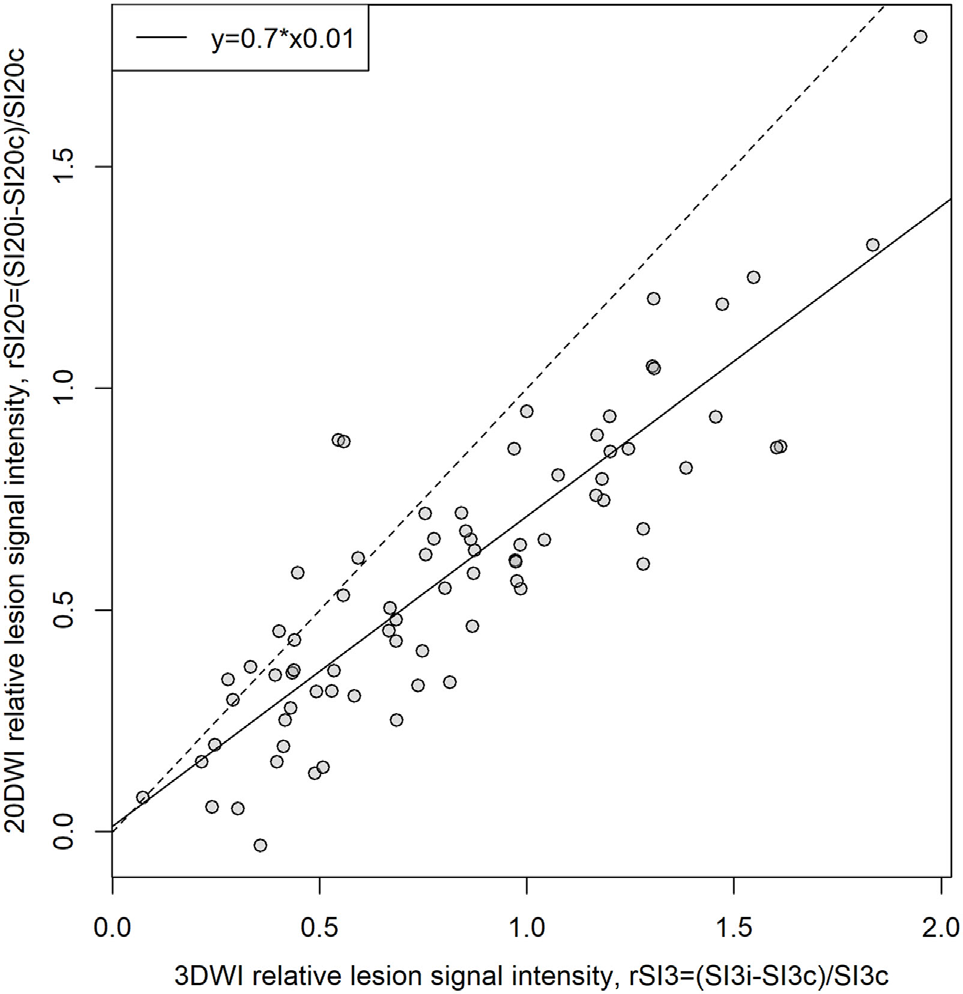
Comparison of relative lesion signal intensity (rSI) on 3-gradient direction DWI (3DWI) and 20DWI, *N* = 76. rSI is the normalized difference between signal intensities in lesions and contralateral homologous normal tissue, i, ipsilateral (lesion); c, contralateral. 3- and 20DWI sequences’ relative lesion signal intensity shows linear correlation. Best linear model fit has 0.7 incline implying that 3DWI has 30% higher relative lesion signal intensity than 20DWI. The dashed reference line has slope 1 indicating where rSI3 = rSI20.

The relative signal intensity on 3- and 20DWI was linearly correlated (Figure 4), and the relative lesion signal intensity was higher on 3DWI than 20DWI [median (IQR) 0.77 (0.48–1.17) versus 0.58 (0.34–0.81), *P* = 0.0006]. 20DWI’s median relative signal variation was systematically lower than 3DWI’s [median (IQR) rSI20/rSI3: 0.70 (0.59–0.84)].

**Figure 5:**
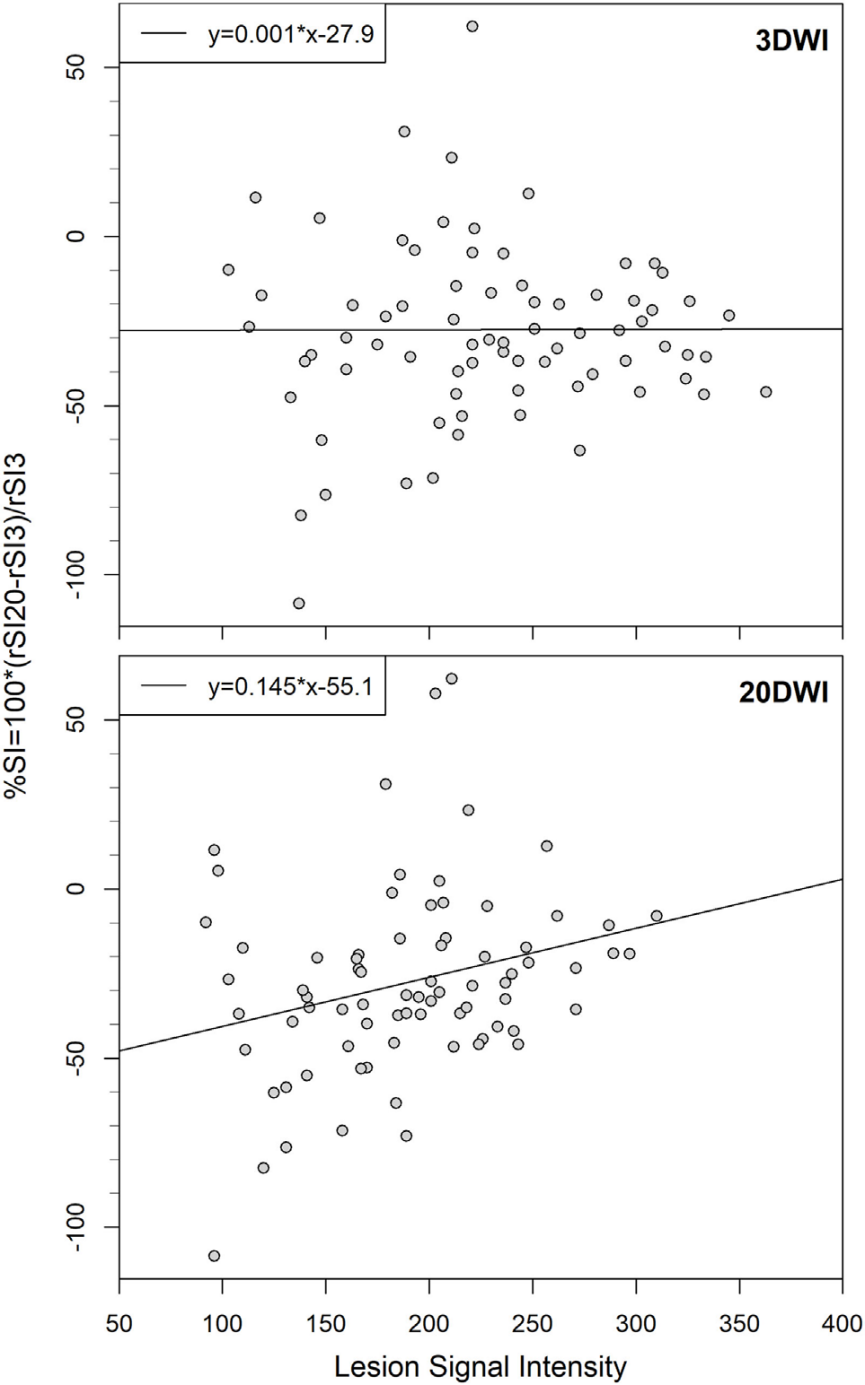
Comparison of difference in relative signal intensity (%SI) to absolute signal intensities on 3-gradient direction DWI (3DWI)- and 20DWI, linear model regression lines with equations, *N* = 76. Difference in relative signal intensity is uniform for absolute 3DWI lesion signal intensities, but lower for low-signal lesions on 20DWI.

**Figure 6:**
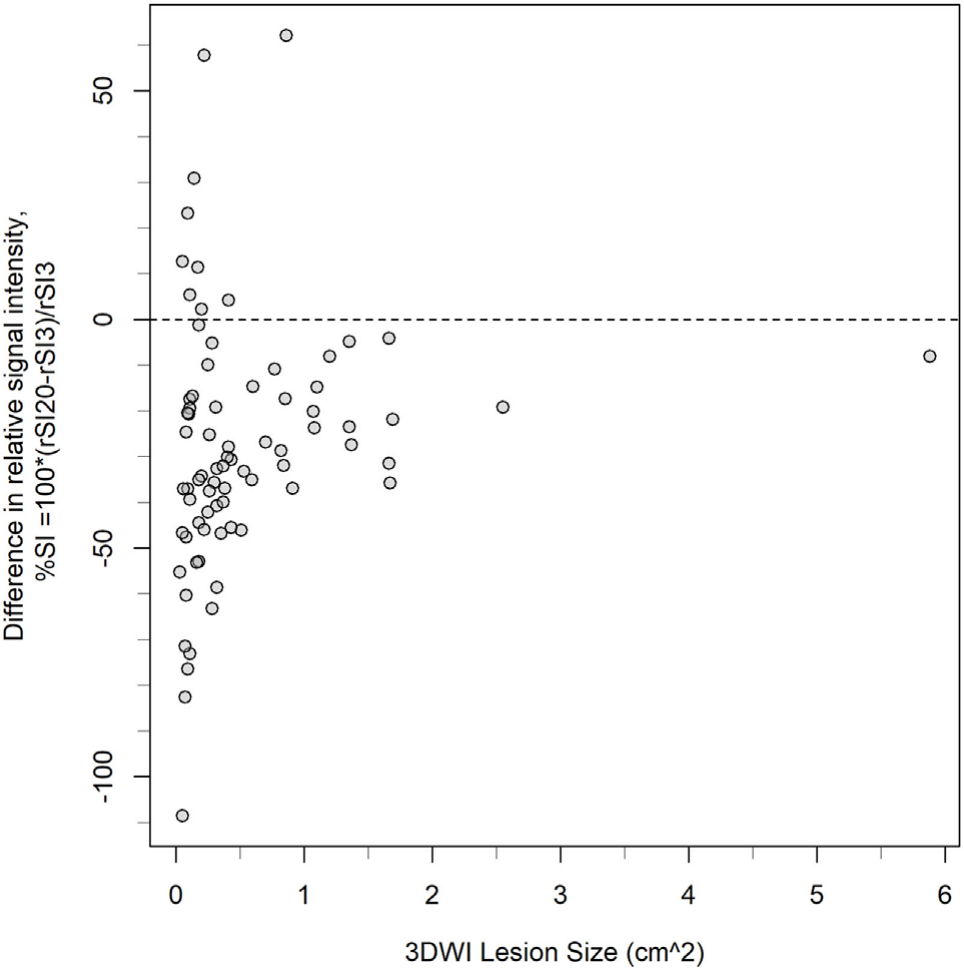
Distribution of lesions according to size measured on 3-gradient direction DWI (3DWI), and the two sequences’ difference in relative signal intensity (%SI), *N* = 76. The dashed reference line indicates where rSI3 = rSI20. Small lesions show highest variation in the two sequences’ difference in relative signal intensity (%SI), and predominantly highest relative signal intensity on 3DWI (i.e., %SI is negative).

The difference in relative signal intensity (%SI) was positively correlated to absolute 20DWI lesion SI (Figure 5, lower panel, Spearman’s rho = 0.28, *P* = 0.01). That is, 20DWI showed increased relative signal intensity on high-contrast lesions, and lower contrast for lesions with lower absolute SI. For 3DWI there was no correlation between the difference in relative signal intensity (%SI) and absolute 3DWI lesion SI (Figure 5, upper panel, rho = −0.0088), nor relative 3DWI lesion signal intensity (rSI_3_, rho = −0.0837). Small lesions showed predominantly highest SI on 3DWI (Figure 6).

### Final Infarct Size Estimate

**Figure 7:**
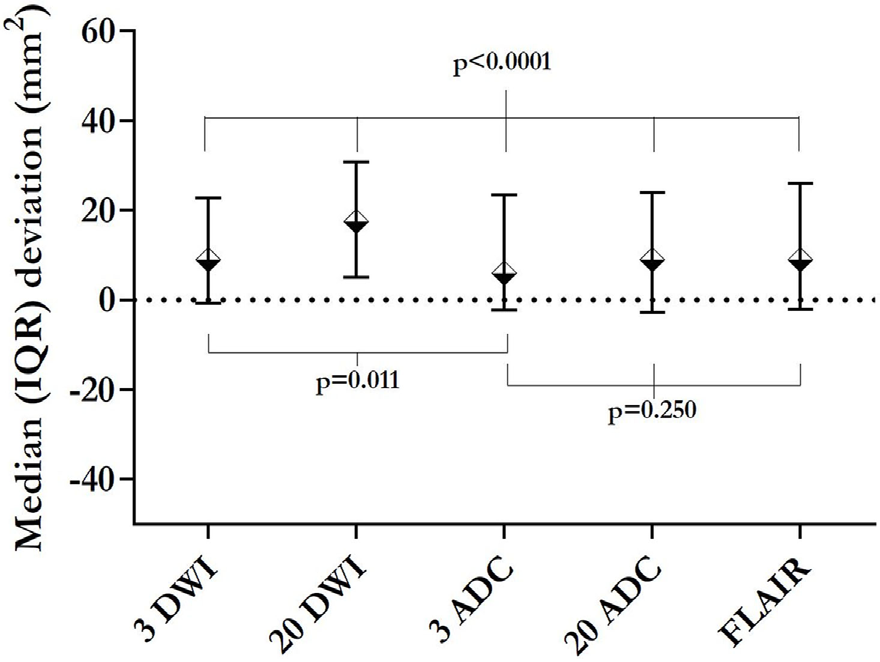
Initial lesions’ median deviation from 8-week infarction area. This figure presents the median deviation between the initial lesion area on the respective sequences, and the area of the final infarction on 8-week magnetic resonance imaging. The smallest deviation (initial 3ADC) is significantly smaller than the deviation yielded by 3-gradient direction DWI (3DWI) or 20DWI. 20ADC and fluid attenuated inversion recovery (FLAIR) yielded deviations not significantly different from 3ADC.

**Figure 8:**
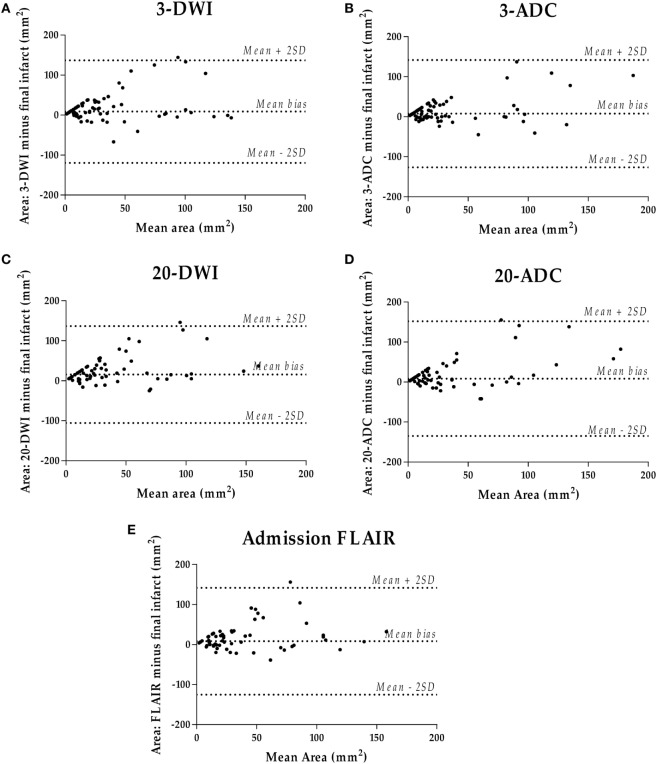
Bland–Altman diagrams per initial sequence **(A–E)** comparing initial lesion size with 8-week infarct size.

There was a significant difference between the different MRI sequences’ ability to estimate the size of the final infarct (*P* < 0.0001). Figure 7 shows that areas measured on subacute 3ADC have lowest median (IQR) deviation compared with the final infarct size [7 (−2.5 to 26) mm^2^]. This was significantly lower than 3DWI (*P* = 0.011) and 20DWI (*P* < 0.001). However, no statistical difference was found between the median deviations on 3ADC, 20ADC, and FLAIR. Complete Bland–Altman diagrams with mean bias are shown in Figure 8.

## Discussion

This study compared fast standard vendor diffusion protocols for mild ischemia detection and characterization in clinical workflow. The faster 3DWI detected more lesions than 20DWI, and 20DWI lesions were often blurred. 3DWI had higher measured and perceived lesion SI. Neither perceived, nor measured lesion brightness was independently associated with persistent infarction, only lesion size. 3ADC in plane area gave the closest prediction of final infarct size, although not statistically better than 20ADC. 20DWI did not add clinically relevant information in a subacute population with TIA.

This study’s main limitations include the small size of TIA-related lesions, lower 20DWI resolution and number of *k*-space averages. For T2-FLAIR low-contrast conditions, and gradual lesion SI increase over time, render quantitative measurements and interobserver study challenging. Challenging is also the subacute time window where small lesions without ADC confirmation, but beginning FLAIR-visibility, are difficult to differentiate from chronic lesions’ T2 shine-though. Therefore, we designed the study to be mainly dichotomous, denoting visible presence or absence of an ischemic lesion, and matched symptoms and lesion site. All patients underwent standardized imaging, and analysis was performed on specialist level, to ensure highest possible homogeneity. Our population was not consecutive, as inclusion was based on informed consent, and presumably showed a higher lesion rate than a consecutive cohort. A potential limitation is the lack of blinding between sequences, but this was not feasible, due to the need for certain lesion identification and comparison. Also, perfusion images were not analyzed in this context.

Clinical diagnosis of these small lesions is valuable, as their presence is correlated to increased morbidity and mortality (38). The previously reported improved lesion detection on a 15-gradient direction DWI compared with 3-gradient DWI (30), used a 3DWI with lower in plane resolution compared with ours. The diversity in 3DWI sequences used, may explain part of the reported variation in TIA populations’ DWI-positivity rates.

Small lesion imaging combines the challenge of high resolution with short acquisition time to limit motion artifacts. Here, we used standard sequences with vendor’s best compromise for resolution. Measuring small lesions, relative to the achieved voxel resolution, one may encounter a relative large fractional measurement error, simply due to small size, and relatively large error margins for small lesions (volume <5 mL) have been reported (39).

The gradual FLAIR SI increase over time (37, 40) is a challenge, especially for small lesions. The interrater agreement was lower for FLAIR than DWI, in a study of DWI–FLAIR mismatch (40). While developed FLAIR high signal showed good interrater agreement, subtler changes showed only moderate agreement (kappa 0.59) (37) and were challenging for clinicians (41). Addition of semiautomated lesion measurement did not improve visual lesion assessment and interrater agreement (36).

The discrepancy between clear clinical stroke or TIA presentation and diffusion-negativity has been widely reported (12, 42), and the more advanced 20DWI sequence, used in this study, did not add further information. On the contrary, 3DWI found two more lesions than 20DWI. 20DWI was prone to blurring, presumably due to lower resolution and fewer *k*-space averages, and possibly intrinsic noise removing postprocessing, and 3ADC area showed better correlation to the resulting infarction area. This study’s findings concur with reservations about TSI subgrouping of TIA patients, as opposed to those without initial DWI lesion. Thus, the best tools available seem to be clinical decision scores, which also take DWI-positivity into account, but still consider TIA patients as one common patient group, as, e.g., ABCD3-I.

This study ran under standardized conditions in a subacute clinical setting, focusing on optimizing high lesion detectability, acceptable scan time and standardized image interpretation after a predefined chart from the PACS alone, without external postprocessing software. It describes a clinical setting where the MRI findings must be both fast to interpret, and the MRI sequences must be equally fast to obtain. Our diffusion protocols had different scan parameters, 20DWI had lower resolution and lower averages in *k*-space to achieve acceptable scan time. These factors may explain lower signal-to-noise ratio and correspondingly lower small lesion detection rates, and lower lesion contrast for low-signal lesions in 20DWI compared with 3DWI, and no improvement in final lesion size projection. Variation in time from ictus to initial MRI may explain why initial T2-FLAIR did not show better lesion size projection (37, 40). We chose to investigate lesion detection, viewing fractional anisotropy ratios as potentially useful to assess tissue damage in the aftermath of ischemia, but not in acute lesion detection, since these measurements require a well-defined and stable lesion area.

Future research holds the short-term challenge of imaging protocol optimization for speed, diagnostic certainty and accessibility. Long-term challenges are clear definitions of diagnostic entities in the spectrum of acute brain ischemia.

## Conclusion

3-Gradient direction DWI detected more lesions and showed higher lesion contrast, regardless of lesion size or absolute SI, than 20DWI. 20DWI was frequently blurred. Risk of persistent infarction related to lesion size.

## Ethics Statement

This study was carried out in accordance with the recommendations of the National Committee of Biomedical Research Ethics with written informed consent from all subjects. All subjects gave written informed consent in accordance with the Declaration of Helsinki. The protocol was approved by the National Committee of Biomedical Research Ethics (H-1-2011-75).

## Author Contributions

IH, HC, and AC conceived and designed the study. All the authors were involved in data acquisition, reviewed and edited the manuscript, and approved the final version of the manuscript. KÆ, JM, PM, SR, MF, and HC were involved in patient inclusion. IH, CO, JN, AC, and HC analyzed and interpreted data. IH wrote the first draft.

## Conflict of Interest Statement

CO holds research grants from the Velux-foundation, Bispebjerg University Hospital, University of Copenhagen, Axel Muusfeldts Foundation, and Danish Medical Association. None of these were designated for this study.

## Abbreviations

3DWI, 3-gradient direction diffusion-weighted imaging; 20DWI, 20-gradient direction diffusion-weighted imaging; ADC, apparent diffusion coefficient; DTI, diffusion tensor imaging; DWI, diffusion-weighted imaging; FLAIR, fluid attenuated inversion recovery; FOV, field of view; IQR, interquartile range; MRI, magnetic resonance imaging; SI, signal intensity; SNR, signal-to-noise ratio; TE, echo time; TIA, transient ischemic attack; TR, repetition time; TSI, transitory symptoms with infarction.
